# Comparison of Delivery Methods in Phage Therapy against *Flavobacterium columnare* Infections in Rainbow Trout

**DOI:** 10.3390/antibiotics10080914

**Published:** 2021-07-27

**Authors:** Heidi M. T. Kunttu, Anniina Runtuvuori-Salmela, Mathias Middelboe, Jason Clark, Lotta-Riina Sundberg

**Affiliations:** 1 Nanoscience Center, Department of Biological and Environmental Science, University of Jyväskylä, FI-40014 Jyväskylä, Finland; Heidi.kunttu@jyu.fi (H.M.T.K.); anniina.runtuvuori@jyu.fi (A.R.-S.); 2 Marine Biological Section, Department of Biology, University of Copenhagen, DK-3000 Helsingør, Denmark; mmiddelboe@bio.ku.dk; 3 FixedPhage Ltd., Glasgow G20 0SP, UK; jason.clark@fixed-phage.com

**Keywords:** aquaculture, bacteriophage, bacterial infection, columnaris disease, *Flavobacterium columnare*, phage therapy, phage delivery, rainbow trout, treatment, virulence

## Abstract

Viruses of bacteria, bacteriophages, specifically infect their bacterial hosts with minimal effects on the surrounding microbiota. They have the potential to be used in the prevention and treatment of bacterial infections, including in the field of food production. In aquaculture settings, disease-causing bacteria are often transmitted through the water body, providing several applications for phage-based targeting of pathogens, in the rearing environment, and in the fish. We tested delivery of phages by different methods (via baths, in phage-coated material, and via oral delivery in feed) to prevent and treat *Flavobacterium columnare* infections in rainbow trout fry using three phages (FCOV-S1, FCOV-F2, and FCL-2) and their hosts (FCO-S1, FCO-F2, and B185, respectively). Bath treatments given before bacterial infection and at the onset of the disease symptoms were the most efficient way to prevent *F. columnare* infections in rainbow trout, possibly due to the external nature of the disease. In a flow-through system, the presence of phage-coated plastic sheets delayed the onset of the disease. The oral administration of phages first increased disease progression, although total mortality was lower at the end of the experiment. When analysed for shelf-life, phage titers remained highest when maintained in bacterial culture media and in sterile lake water. Our results show that successful phage therapy treatment in the aquaculture setting requires optimisation of phage delivery methods in vivo.

## 1. Introduction

The aquaculture industry is under increasing pressure to produce food for the constantly growing world population. Intensive fish farming practices are required to provide increasing quantities of high-quality dietary protein. However, this creates favourable conditions for outbreaks of infectious diseases. To combat bacterial infections, vast amounts of antibiotics are used in aquaculture, leading to antibiotic leakage to natural waters [1,2]. Due to the increased risk of development of antibiotic resistance among bacteria at farms and in the environment, alternative ways of treating and preventing bacterial infections are urgently needed.

In the recent years, the need for alternatives to antibiotics has given a new push to phage therapy research in both human and veterinary medicine [3]. Viruses that infect bacteria, bacteriophages, were discovered at the beginning of the 20th century, and then quickly used to treat bacterial infections, i.e., phage therapy. Since the discovery of antibiotics, interest in the development of methods using phages as therapeutic agents decreased [4,5,6].

In the field of aquaculture, the lack of efficient vaccines against some diseases threatening fish fry presents a challenge to preventing infectious diseases [7]. Phage therapy has been studied as an alternative for antibiotics to prevent and cure bacterial diseases. Promising results have been obtained, e.g., for *Aeromonas salmonicida* infections in brook trout (*Salvelinus fontinalis*) [8] and Senegalese sole (*Solea senegalensis*) [9], *Flavobacterium columnare* infections in zebra fish (*Danio rerio*) and rainbow trout [10,11], *Flavobacterium psychrophilum* infections in rainbow trout [11], *Pseudomonas plecoglossicida* infections in ayu (*Plecoglossus altivelis*) [12,13], and *Vibrio anguillarum* infection in Atlantic salmon (*Salmo salar*) [14].

There are several key considerations when practically applying phage therapy in aquaculture to attain the desired outcome, reduction or elimination of mortality caused by bacterial infection. First, the timing and frequency of phage delivery should be planned carefully, keeping in mind the virulence characteristics of the bacterial pathogen and nature of the outbreaks [7]. Second, the optimal route of phage administration must be identified for each bacterial infection [7,15]. Since pathogenic bacteria have different infection routes (internal or external), it is clear that some bacterial infections are treatable by phages given orally, via intraperitoneal (i.p.) or intramuscular (i.m.) route, whereas others require bath or topical application. Third, an appropriate dose of phages must be determined, which will depend on the expected number of target bacteria, i.e., multiplicity of infection (MOI) is of great importance [7,15,16]. The principle would be to keep the MOI as low as possible while still allowing sufficient bacterial clearance to prevent or stop an outbreak, since using unnecessarily high phage doses is not cost effective for fish farmers. Furthermore, long-lasting stability and resultant infectivity are essential features for phages to be used as therapeutics [16]. Optimal storage conditions are phage-dependent and should be investigated for each potential therapeutic agent.

In aquaculture systems, the real situation often is much more complicated than “one pathogen—one disease”. Fish may suffer from multiple simultaneous infections caused by many bacterial species, or co-infecting parasites, which can influence the outcome of the disease [17,18,19]. Several bacterial strains can be present during disease outbreaks [20], causing further challenges for phage therapy. Since most of the phages usually are very host-specific, infecting only one bacterial species or subspecies, mixture of multiple phages with overlapping host ranges are normally used (reviewed by, e.g., [21,22]).

*Flavobacterium columnare* causes epidermal infections in farmed fish worldwide and is associated with repeated antibiotic treatments and high mortality [20,23,24,25]. *F. columnare* isolates from Finland can be divided into different genetic groups A–H [26,27,28], and phages infecting *F. columnare* are very host-specific, usually infecting only one genetic group [29]. *F. columnare* belonging to several genetic groups can be isolated from the same outbreak and even from the same rearing tank [20,26]. This means that in order to tackle columnaris infection at the farms, phages infecting several genetic groups should be applied simultaneously.

While it has been shown that phages can significantly reduce mortality of zebra fish and rainbow trout in laboratory-induced columnaris disease [10,30], optimal phage mixture, dose, and delivery methods of *F. columnare* phages have remained unclear. In this study, we addressed these issues in three phage therapy experiments with rainbow trout fry, which were treated with phage either prophylactically or after bacterial exposure. In addition, the shelf-life of phages used for treating the fish was studied in different storage conditions. Our results show, that *F. columnare* phages maintain high infectivity in either lake water or *F. columnare*-specific growth medium, and that the most effective phage therapy method against columnaris infections in rainbow trout is phage bath-exposure immediately after the first symptoms of the disease appear in the fish population.

## 2. Results

### 2.1. Phage Therapy Experiment I: Individual Phages and Phage Mixes

**Figure 1:**
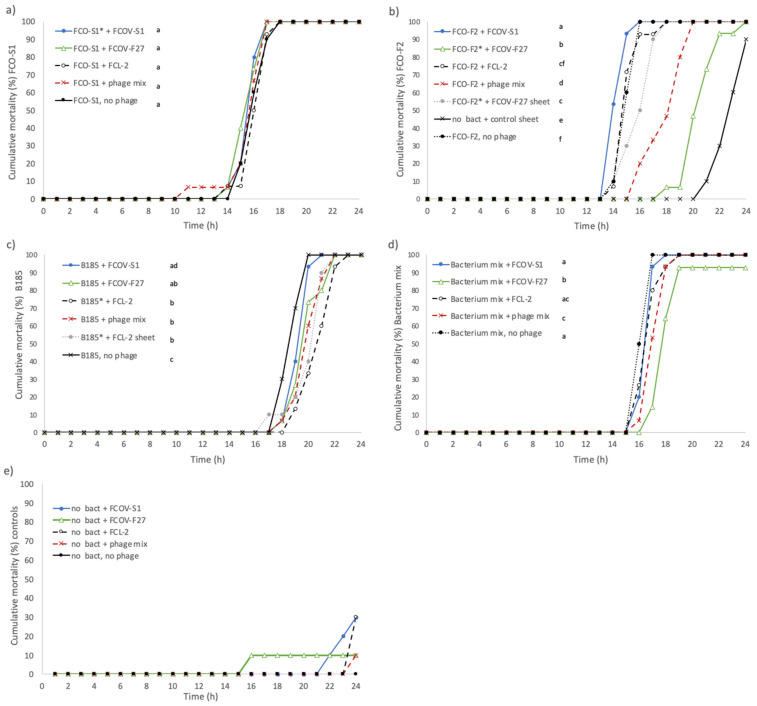
Experiment I: Cumulative mortalities (%) of rainbow trout fry infected with *Flavobacterium columnare* isolates (**a**) FCO-S1, (**b**) FCO-F2, (**c**) B185, and (**d**) their mix, and treated with *F. columnare* phages FCOV-S1, FCOV-F27, and FCL-2, their mix and plastic sheets coated with FCOV-F27 and FCL-2. Mortality related to control treatments without bacterial infections are presented in panel (**e**), except for the plastic control sheet (no phage coating) presented in panel (**b**). The bacterial host of the phage is indicated by an asterisk in each panel. Different lower-case letters a-f indicate statistical difference (*p* < 0.05, Kaplan–Meier survival analysis, log rank pairwise comparisons, Mantel–Cox) between the treatment groups (*n* = 15 rainbow trout in treatments receiving both phage and bacteria, and 10 fish in sheet and control treatments, total *n* of fish = 360).

In the first experiment, we tested how the presence of individual phages and phage mixes influence *F. columnare* infection in rainbow trout. Three bacterial strains (FCO-S1, FCO-F2, and B185) and their phages (FCOV-S1, FCOV-F27, and FCL-2, respectively) were used at MOI 1 [10,31]. Fish in negative control groups, without bacterial challenge, survived significantly longer than all other treatments where bacteria were present (Figure 1a). Compared to the fish having received only bacterial infection, the phage treatments did not have any effect on mortality in fish infected with *F. columnare* strain FCO-S1 (Figure 1a).

Among the fish infected with strain FCO-F2, phage treatments caused a significant decrease in progression of the mortality rates (*p* < 0.05), although the endpoint mortality reached 100% in all cases. Mortality was slowed down the most by phage FCOV-F27 (infecting FCO-F2), which caused approximately a five-hour delay in reaching 50% mortality compared to the phage-free control. With phage mix (a combination of all 3 phages) 50% mortality was reached around 3 h later than in the control, and in FCOV-F27-coated sheet treatment one hour later (Figure 1b). Treatment with phage FCL-2 did not have significant effect; phage FCOV-S1 increased the mortality rate compared to the fish with no phage treatment.

In fish infected with bacterial strain B185, the mortality rate decreased similarly in all other phage treatments except FCOV-S1, which did not have any effect (Figure 1c). In infections induced by mixtures of bacteria, cumulative mortality rate was decreased the most by FCOV-F27 and then by phage mix treatment (Figure 1d). Treatments with FCOV-S1 and FCL-2 did not affect the mortality rates when compared to the fish with only bacterial infection. On the other hand, mortality rates of the fish with FCOV-F27 and FCL-2 treatments did not differ from each other. Among fish having received only phage treatments, cumulative mortality rates did not differ from that of negative control fish with no bacterial infection and phage treatment. Plastic control sheet treatment, however, caused a higher mortality rate than the only phage and negative control treatments. *F. columnare* was isolated from all fish with bacterial infection but not from fish in the infection control (no bacteria).

**Figure 2:**
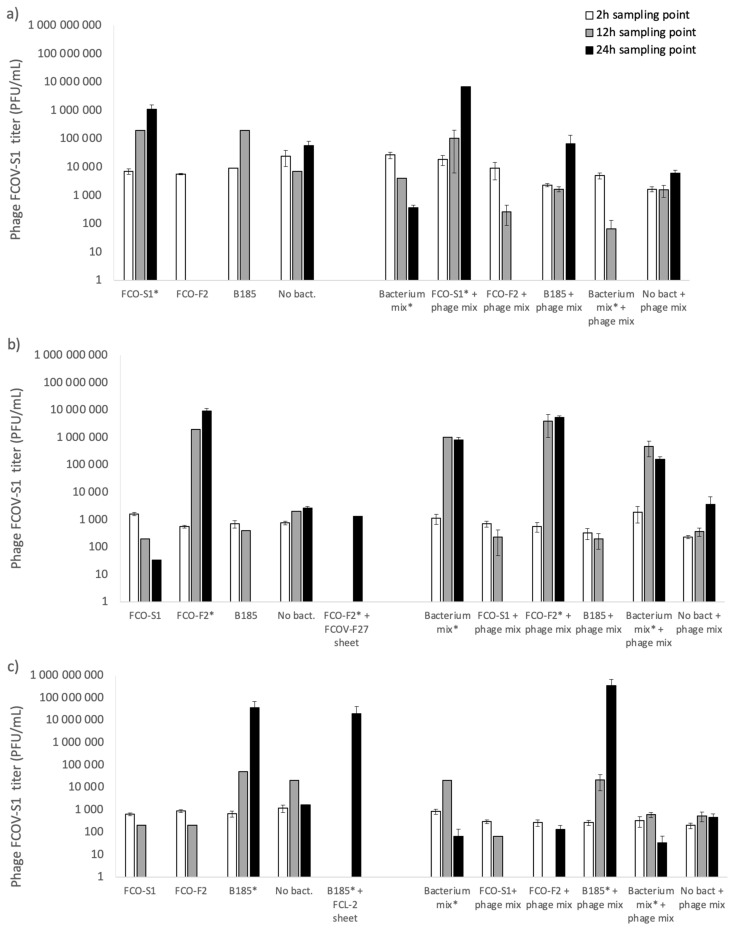
Mean titers (PFU mL^−1^) of phages (**a**) FCOV-S1, (**b**) FCOV-F27, and (**c**) FCL-2 in water samples during bacterial infection of rainbow trout with *F. columnare* strains FCO-S1, FCO-F2, B185, and their mixes. Phage titers were monitored at 0 h (white bars), 12 h (dark grey bars), and 22 h (black bars). Error bars indicate standard error of mean (not available for all 12 h samples). The presence of the phage host is indicated by an asterisk (*).

The number of phages in aquarium water was monitored in all treatments where they were added (Figure 2). From samples taken at 0 h and 12 h, phages were present in all the treatments with a bacterial infection (except phage FCOV-S1, which was not detected in FCO-F2 infection). At the end of the experiment (24 h), phages were only detected in samples where their own host was present, either alone or in a mixture with other bacteria.

Phage titers generally increased in treatments where the host bacterium was present, indicating successful phage replication during the bacterial infection. Titers of phages FCOV-F27 and FCL-2 increased by 3–4 logs (from the initial 5 × 10^3^ PFU mL^−1^) when fish were infected with their host bacteria alone or if it was present in the bacterial mixture. With phage FCOV-S1, the titer increase was more subtle (2 logs) and was not observed in the bacterium mix. If phages were delivered as a mix (total MOI 1), individual phage titers increased most efficiently in single-host bacterium infections, but if the infection was done with the bacterial mixture, only FCOV-F27 titers increased (4 logs).

### 2.2. Phage Therapy Experiment II: Effect of Phage Dose

**Figure 3:**
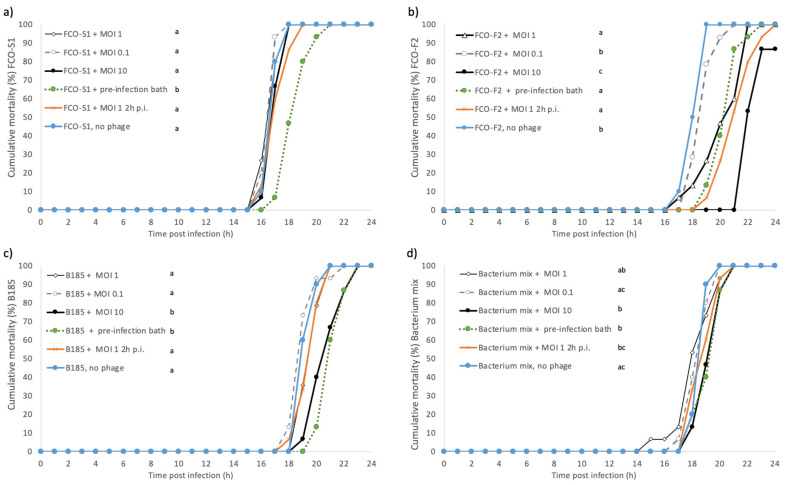
Experiment II: Cumulative mortalities (%) of rainbow trout fry infected with *Flavobacterium columnare* isolates (**a**) FCO-S1, (**b**) FCO-F2 (**c**) B185, and (**d**) their mix, and treated with a mix of phages FCOV-S1, FCO-F27, and FCL-2. Phage mixes were added at the same time as bacteria (0 h) at MOI 1, 0.1, and 10, 2 h post-bacterial infection at MOI 1 (MOI 1 2 h post-infection, p.i.) or given as a 2-h bath exposure at MOI 1 before adding the bacteria (pre-infection bath). Different lower-case letters a-c indicate statistical difference (*p* < 0.05, Kaplan–Meier survival analysis, log rank pairwise comparisons, Mantel–Cox) between the treatment groups (*n* = 15 in phage treatments, *n* = 10 in controls, total *n* = 400).

In this experiment, the effect of phage mix dose (MOI 10, 1, and 0.1) when given at the time of bacterial infection was tested. The same bacterial strains (FCO-S1, FCO-F2, and B185), and their phages (FCOV-S1, FCOV-F27, and FCL-2, respectively) were used as described previously. With MOI 1, the timing of phage application was also examined; phage treatment was given before or 2 h after bacterial exposure. The only treatment that decreased the cumulative mortality rate of FCO-S1-infected fish was a pre-infection phage bath (Figure 3a). Pre-infection bath treatments were also the most effective in reducing mortality rate in fish infected with B185 (Figure 3c) and the bacterial mix (Figure 3d). In FCO-F2 infected fish treated with phage mix at MOI 10, the overall mortality was reduced to 86.7% (compared with 100% in all other treatments) (Figure 3b), and this treatment also had the strongest effect on cumulative mortality rates. Other phage treatments, excluding MOI 0.1, significantly decreased the mortality rate of FCO-F2-infected fish.

There was no mortality among negative control fish (no bacterial infection and phage treatment) or among fish in pre-infection phage bath treatment without bacterial infection (results not shown). However, only-phage mix treatments (no bacteria added) with MOI 1, MOI 0.1, MOI 10, and post-infection bath MOI 1 2 h caused reduced mortality, with mortality rates of 90.0, 60.0, 60.0, and 20.0%, respectively. In other treatments without bacterial infection (results not shown), the cumulative mortality rates did not differ between phage-mix-only treatments, with MOI 1, 0.1, and 10, between pre-infection bath, post-infection bath, and negative control, and on the other hand between MOI 0.1 0 h, MOI 10 0 h, and post-infection (MOI 1 2 h) phage treatments. *F. columnare* could be cultured from the groups with dead fish with bacterial infection but were not detected in control fish without bacterial exposure.

### 2.3. Phage Therapy Experiment III: Phage Delivery in Flow-Through System

**Figure 4:**
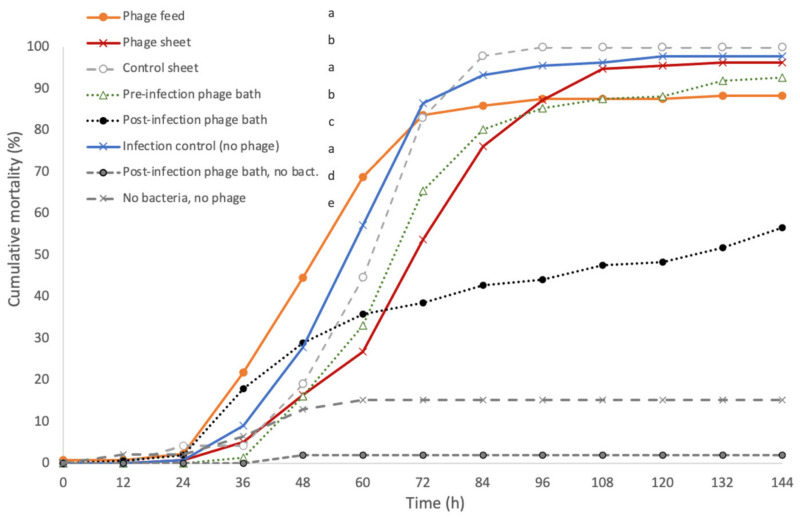
Experiment III: Cumulative mortalities (%) of rainbow trout fry infected with a mix of *Flavobacterium columnare* isolates FCO-F2 and B185. Rainbow trout were kept in three replicate aquaria per treatment (48 fish each) except for controls, which included only a single aquarium (total *n* of fish in experiment = 960). The fish were fed 7 days before the infection with phage mix-coated (FCOV-F27 and FCL-2) feed (phage feed), kept with the phage mix-coated sheet (phage sheet), or a control sheet without phage mix (control sheet) during the whole experiment starting from 7 days before bacterial infection, or treated with a 2-h phage mix bath one day before bacterial infection (pre-infection phage bath) and right after the first symptoms of columnaris disease appeared after the bacterial infection (post-infection phage bath). Negative controls for pre-infection phage bath and phage feed did not cause any mortality and are not shown in the graph. Different lower-case letters indicate statistical difference (*p* < 0.05, Kaplan–Meier survival analysis, log rank pairwise comparisons, Mantel–Cox) between the treatment groups.

The third experiment was done in a more realistic flow-through system with rainbow trout populations. Here, we tested phage delivery via feed, plastic sheets with immobilised phages, or baths (before and after bacterial infection, or as a treatment at the start of the outbreak). Mortality was lowest (56.5%) among fish that received a 2-h phage treatment when the first symptoms of columnaris disease appeared (post-infection phage bath) (Figure 4). A 2-h phage mix bath treatment before bacterial infection (pre-infection phage bath) slowed down the cumulative mortality (endpoint mortality 92.7%), which did not differ from that of fish exposed to phage delivery by coating on plastic sheets. Surprisingly, among the fish receiving the phage orally via feed, the cumulative mortality increased the fastest during the first two days but stabilised after that reaching the endpoint mortality of 88.3%. However, cumulative mortality of phage-fed fish did not differ statistically from that of infection control (bacteria only) or control sheet (with no phage attached). Phage-coated sheet treatment somewhat slowed down the mortality rate compared to the infection control and control sheet treatments (*p* < 0.05).

Bacterial infection caused 96–100% mortality among fish in all treatments: phage sheet (96.3%), infection control (97.7%), and control sheet (100%) (Figure 4).

No mortality was observed in the bacterium-free pre-infection phage bath and phage feed treatments, but some background mortality was observed in the post-infection phage bath control and in phage-free control. *F. columnare* was isolated from all the dead fish exposed to bacteria. Phages, however, were only detected in three out of nine sampled fish fed for one week with phage-coated feed (Table S1): FCOV-F27 was detected in skin mucus and intestine of two fish and from kidney of one fish, and FCL-2 from skin mucus of two and intestine of three fish. Both phages were detected in the water only in two phage bath-treated aquaria in samples taken at the end of the experiment.

### 2.4. Shelf-Life of Phages in Different Storage Conditions

Two experiments focusing on the shelf-life of phages in different storage conditions were conducted. When testing suitability of different buffers for phage preservation, all phages maintained the highest infectivity in Shieh medium (Figure S1). In other buffers, the infectivity of phages infecting genetic groups C and G decreased toward the end of the experiment, although higher titers at some testing points were observed in certain buffers. The variability in results from different sampling points are most likely caused by variability in bacterial growth during titration.

**Figure 5:**
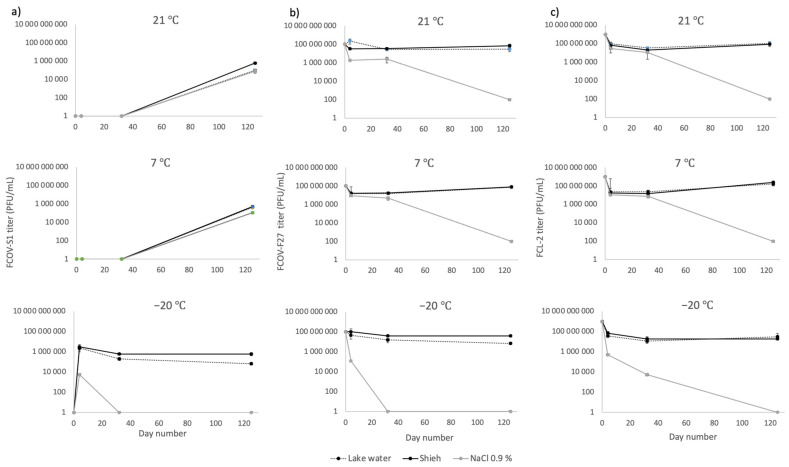
Shelf-life, determined as mean plaque forming units (PFU) mL^−1^, of Flavobacterium columnare phages (**a**) FCOV-S1, (**b**) FCOV-F27, and (**c**) FCL-2 at different storage temperatures and in different media. Error bars indicate standard error of mean. Number of replicate measurements was 3 for lake water and 2 for Shieh medium and NaCl 0.9%.

In a second experiment, phage shelf-life was tested at different temperatures (−20 °C, 7 °C, and 20 °C) using Shieh medium, lake water (from Lake Jyväsjärvi), and 0.9% NaCl as preservation media. In general, phage survival was best in Shieh medium and in lake water (Figure 5). FCOV-F27 and FCL-2 titers did not drop more than approximately 1 log during the 125 days in all conditions if stored in Shieh medium or lake water. However, in 0.9% NaCl, the infectivity of phages remained stable at room temperature and at 7 °C up to 32 days (second sampling timepoint), after which the phage titers started to decrease, leading to a titer of approximately 10^2^ PFU mL^−1^ on day 125 (Figure 5). FCOV-S1 titers could not be determined at room temperature or at 7 °C during the first sampling point days due to poor growth of the host, but high titers (around 10^6^ PFU mL^−1^) in all media were detected on day 125. All three phages could be revived from 149 and 468 days of dry conditions (data not shown).

## 3. Discussion

As pathogen-specific, self-templating, self-enriching, and naturally occurring biological entities, lytic phages are appealing tools for biocontrol in aquaculture systems. Indeed, phages can be used in treatment of existing disease outbreaks, but also in disinfection of water and biofilms to prevent disease development and transmission. Phages have been observed to effectively reduce bacterial growth in in vitro studies with fish pathogens *A. salmonicida* [32,33] and *Vibrio parahaemolyticus* [34]. Environmentally transmitting pathogens often have heterogeneous populations where several genetically different strains co-exist, so the development of phage mixtures with coverage of the desired target strains is essential. Furthermore, optimal phage dosing and delivery methods are critical for success of both preventive and treatment approaches. Choosing the best delivery approach is especially important for aquaculture settings, where the disease agents can spread via water or fish faeces and form biofilms on rearing equipment. In this study, we tested different approaches to target *F. columnare* strains. Using rainbow trout fry as an infection model, we tested different delivery methods, such as bathing before and after infections, and oral administration of phages via feed. Our results showed mortality related to columnaris disease was reduced most efficiently by phage bath treatment, applied to the fish when the first disease symptoms had appeared, although other delivery methods also slowed down the progression of the disease.

From the perspective of choosing phages to include in a mixture, our results show that with *F. columnare* the protective effect of phages depends on their infection efficiency in their specific host, but that no clear pattern could be seen with the applied phage-to-bacterium doses (multiplicities of infection) tested. Timing of phage treatment (at the same time or 2 h after the bacterial infection) or delivery (bath or constant) also did not substantially affect outcomes in this study. Mortality related to mixed bacterial infection was slowed by both phage mix (MOI 1 given 2 h after bacterial exposure, or MOI 10 at the same time with bacteria) and FCOV-F27 alone. Since the bacterial strain FCO-F2 was the most virulent of the three strains, the efficiency of phage in prevention of fish mortality indicates FCOV-F27 has a strong lytic activity during bacterial infection. A phage mix containing all three phages (FCOV-S1, -F27, and FCL-2) slowed the mortality rate when fish were infected with bacterial strains B185 and FCO-F2, and similar results were obtained when fish infected with these strains were treated with their specific phage. Surprisingly, FCO-S1 infection was not affected by its own phage FCOV-S1, and this phage-bacterium pair was not used in further experiments.

When different phage doses were studied, it was found that an MOI of 0.1 did not provide protection against the disease in any of the cases. MOI 10 (given at 0 h) had the strongest effect on cumulative mortality rate in fish infected with FCO-F2 and B185 (Figure 3c). In previous experiments with FCL-2 and B185, no clear difference was found in the survival of zebra fish between MOI 1 and MOI 100 (continuous phage exposure) or in rainbow trout between MOI 1 and MOI 10 (phage exposure by bathing) [10]. However, it should be noted that here our first experiments were done in stagnant water, where a high bacterial dose was maintained. This type of condition does not completely reflect to disease dynamics happening at fish farms. Subsequently, phage treatments were also tested in flow-through conditions. Furthermore, compared to antibiotics, where the initial dose is central for successful treatment, phages are self-replicating, which changes the phage-to-bacterium ratio when the bacterial hosts are present.

In the continuous bacterial exposure in stagnant water, the only truly effective treatment was a two-hour phage-mixture bath (MOI 1) given before the bacterial infection. In this treatment, fish were pre-colonised with phage, which has shown to be efficient to slow down the onset of disease [30]. Some tailed phages, including *F. columnare* phages, have the capacity to bind to mucin glycoproteins via Ig-binding domains [35,36] and be maintained in the mucosa for up to 7 days [30]. Since *F. columnare* bacteria has a strong chemotaxis towards fish mucosa, preventive phage baths allow replication of phage immediately upon bacterial colonisation, which delays the onset of the disease. In real-life farming systems, the effect of introduced mucosal phages might even be stronger as the bacterial numbers initiating the infection are likely to be lower than in the experimental infection used here. Pre-colonisation of vertebrate hosts with phages has been shown to be efficient also in preventing *Vibrio cholerae* infections in mice [37], and the approach has been used also in humans in the former Soviet Union [38].

Conducting experiments in conditions resembling the real-life rearing environment is important to allow a better understanding of the efficiency of phage treatments. In the flow-through experimental system (experiment III, which most closely resembled the real-world situation) the phage bath treatment significantly reduced fish mortality. The greatest effect was observed when the bath treatment was given when the first symptoms of columnaris disease were observed. Phage bath treatment has previously been shown to decrease and slow down the mortality of *F. columnare* infected rainbow trout [10,30], but in these cases the phage was delivered prior to the symptoms of bacterial infection appearing. Here, phage treatment at the onset of epidemic probably targeted both bacteria replicating in the fish and also those transmitting in the water. Furthermore, it is possible, that the increased replication rate of bacteria on the fish mucosa exposed them to phage infections, leading to efficient reduction of bacterial burden and transmission (see also [30]). Nevertheless, using phage baths as “medication” seems an efficient way to stop progression of the outbreak. It remains to be studied how such treatment influences the development of phage resistance in target bacteria, and if a subsequent phage treatment should include a mixture (“cocktail”) of different phages.

Administration of phages via water is conceptually simple, but in commercial-scale aquaculture the volumes of water requiring phage treatment can be impractically large. Attaching phages on surfaces to provide materials that bioactivate upon contact with the host bacteria is therefore an alternative option to efficiently deliver phages in rearing systems. Here, we tested two approaches of attached phages: plastic sheets and fish feed. Phage-coated plastic sheets delayed fish mortality significantly both in stagnant water (experiment I, Figure 1) and in the flow-through system, although other approaches were more efficient. Nevertheless, the results suggest that immobilisation of phages on aquaculture-relevant surfaces, e.g., biofilters, could be an efficient way to reduce the effect of environmentally transmitting pathogens in inlet and tank water at flow-through fish farms. This would allow maintaining a “biobank” of pathogen-targeting phages that are able to interact with target bacteria in the water and initiate infection cycles to enrich and spread phage progeny in the bacterial population at farms during the rearing cycle. Phage immobilisation techniques have several generalisable downstream applications in biomedicine and food production. For example, phage coating of catheters efficiently prevents *Staphylococcus epidermitis* biofilm formation [39] and prevents *Pseudomonas aeruginosa* in wound dressing [40], and phage coating of food packing materials has been applied to prevent food from spoiling [41].

The effect of prophylactic oral administration of a phage-mix via coated feed was tested against columnaris disease. Interestingly, phage feed did not have a major protective effect on fish mortality, though in experiment III the endpoint mortality was lower than in the control group. However, in the beginning of the experiment mortality increased rapidly in phage-fed groups, resulting in endpoint mortality being higher than in groups having received phage bath treatment after the bacterial infection. In a recent study where a similar phage immobilisation technique on fish feed was used, oral phage delivery did not protect fish from *F. psychrophilum* infection [42]. In other studies, phage-impregnated feed has been shown to have a protective effect against bacterial infections, e.g., in *P. plecoglossicida* infections of ayu [12,43] and *Lactococcus garvieae* infections in yellowtail (*Seriola quinqueradiata*) [44]. The most probable reason for the inefficiency of phage-coated feed against columnaris disease is the external infection route and symptoms of *F. columnare*. The bacterium is probably not cleared by orally applied phages as efficiently as by phages applied from outside the body. While phages were detected in the organs of fish fed phage-immobilised feed, this was at relatively low numbers and transmission of phages through the gut appears to be relatively inefficient.

However, other explanations are worth considering. In our flow-through experiment (experiment III), the feed was coated with a crude phage lysate that would include bacterial debris, possibly containing intra- and extracellular bacterial toxins, which could affect the overall welfare of fish and make them more susceptible to experimental bacterial infection. In this light, it might be possible that the primary immune system of small fish reacts heavily to parenterally delivered phages and this kind of energy allocation weakens their ability to resist bacterial infections, as has been shown with immunostimulant delivery to rainbow trout fry [45]. Phage therapy is known to have variable immunomodulatory effects, e.g., changes in cytokine and C-reactive protein responses, in humans (reviewed by [1]). Immune responses in the fish were not examined in this study, but based on two independent experiments, it is clear, however, that phage exposure via oral route does not improve the recovery of rainbow trout fry from *F. columnare* infection.

All the three phages could be isolated from the aquarium water in all the treatments of experiments I and II (stagnant water experimental system) for 24 h (excluding treatments with other than phage’s own host). However, phages could only be found in intestine, skin mucus, and kidney in a minority of the sampled fish after one-week prophylactic feeding with phage-coated feed in the flow-through experimental system (experiment III). No phages were detected in spleens or in the aquarium water before the bacterial infection in the phage-fed fish. Additionally, no phages were detected from treatments with sheet or bath pre-infection, which contrasts with recent results using *F. columnare* phages that were found to maintain their activity in fish skin mucus for up to one week [30] and in aquarium water for two days [10,30] in flow-through systems. However, in these previous studies, the bacterial infection had been applied before phage sampling, allowing phage replication. Our present results also differ from those observed in rainbow trout using *F. psychrophilum* phages in which phages were not only detected in the intestine and kidney but also from the spleen after one-week feeding [46]. However, when using oral intubation and bathing [46] or intraperitoneal injection [47] as application routes, *F. psychrophilum* phages were cleared from intestine, spleen, and i.p. cavity of rainbow trout after three or four days if no host bacteria were present.

One major criticism against phage therapy is the rapid development of phage resistance. It has been suggested that one way to reduce this issue is the simultaneous use of multiple phages that target different bacterial receptors [21,48,49]. It has been shown in in vitro studies using other aquatic pathogens that, compared to single phages, phage mixtures more efficiently inhibit both the growth and development of phage resistance in *Vibrio* [34] and *A. salmonicida* [32,33]. In this study, only phage mixtures against infections of bacteria belonging to different genetic groups were tested. Thus, in the future, the effect of mixtures with phages infecting one genetic group of bacteria but targeting different receptors should be examined.

Compared to a previous study on the shelf-life of *F. columnare* phage, where FCL-2 maintained high (10^9^–10^10^ PFU mL^−1^) titers for six weeks in modified Shieh medium, Tris-HCl and KH_2_PO_4_ at 6 °C [10], the infectivity of phages (incl. FCL-2) decreased clearly in most of the buffers after one week in the present study. On the other hand, regardless of the temperature (−20 °C, 7 °C, and 21 °C), *F. columnare* phages maintained their infectivity for over four months in Shieh medium and autoclaved lake water. Phages could also be recovered from over 15-month desiccation in Shieh medium (data not shown). Similar results have been obtained in *F. psychrophilum* phages, which could maintain infectivity both after two- and eight-month desiccations on feed pellets [46,47], and when preserved in autoclaved fish pond water [47,50] or *F. psychrophilum* -specific growth medium [50]. Also, in *Vibrio* phages, it was shown that the conditions resembling the natural environment of the phage, i.e., marine aquaculture water, favour phage stability [34].

Our study gives encouraging results for the practical development of phage therapy against *F. columnare* infections in fish farming. We show that bath treatment, especially after the first symptoms of columnaris disease appear in the fish population, is efficient in treating the disease in rainbow trout. This delivery method is most likely successful due to the epidermal nature and environmental transmission of the disease. Furthermore, *F. columnare* phages maintain their infectivity at simple storage conditions.

## 4. Materials and Methods

### 4.1. Bacteria and Phage Isolates

Bacteria and phages used in this study were isolated from water samples collected at Finnish and Swedish fish farms during columnaris outbreaks and have been characterised previously [29] (Table 1 and Table S2). The bacterial isolates FCO-S1, FCO-F2, and B185 were specifically infected by the phages FCOV-S1, FCOV-F27, and FCL-2, respectively.

### 4.2. Bacterial Cultures and Phage Purification

All bacterial and phage cultures mentioned in this article were grown in modified Shieh medium [51], called “Shieh” for simplicity.

*F. columnare* isolates were inoculated from cryopreserved (−80 °C) stocks into Shieh medium and grown for 48 h at 25 °C with 120 rpm agitation. Afterward, subcultures were made in Shieh medium and grown for 24 h at 25 °C with 120 rpm agitation. The optical density (OD) of the bacterial broth suspensions was measured spectrophotometrically at 595 nm and adjusted to give a *F. columnare* concentration of 5 × 10^6^ colony-forming units (CFU) mL^−1^ (based on previously determined OD/CFU relationship).

Unless otherwise mentioned, phage lysates were prepared according to [52], were purified by the polyethylene glycol (PEG)-NaCl-method according to [53], and adjusted to 5 × 10^7^, 5 × 10^6^, and 5 × 10^5^ plaque forming units (PFU) mL^−1^ in Shieh medium.

### 4.3. Fish

Rainbow trout fry were received from a fish farm in central Finland where they had hatched about two months before transferring to the laboratory. Fish were held in bore hole water at 16 °C with constant flow-through and aeration and fed with commercial feed pellets. For the experiments, the water temperature in holding aquaria was increased 0.5–1.0 °C per day up to 25 °C.

### 4.4. Phage Therapy Experiments I and II: Effect of Phage Dose and Delivery Method in Constant Exposure

Rainbow trout fry, averaging 0.73 g in Exp I and 0.71 g in Exp II, were placed individually into 0.75-L aquaria containing 500 mL of aerated, 25 °C bore hole water. In Exp I (Table 2) the fish were divided into 28 aquaria. Bacterial infection with three different strains (see Table 1), either individually or as a mixture, was made by pipetting 0.5 mL of bacterial solution of 5 × 10^6^ CFU mL^−1^, giving a constant infection dose of 5 × 10^3^ CFU mL^−1^. Phage treatment was given at the same time with bacterial infection at a MOI of 1 (final dose 5 × 10^3^ PFU mL^−1^ for each individual phage). In the phage mixture, the dose of each phage was 1/3, resulting to total MOI of 1.

In experiment II (Table 3), fish were divided into 30 treatment groups of ten or 15 fish per group depending on the treatment. Fish were again exposed to three bacterial strains (Table 1) in single infections or as a mix. Phage treatment was given at the same time as bacteria or 2 h after adding the bacteria. For each phage, three doses were used. 0.5 mL phages and their mixtures with titers of 5 × 10^7^, 5 × 10^6^, and 5 × 10^5^ PFU mL^−1^ were added into aquaria, giving a final dose of 5 × 10^4^, 5 × 10^3^, and 5 × 10^2^ PFU mL^−1^, respectively, and multiplicities of infection 10, 1, or 0.1. One group of fish was bath-exposed to a phage mixture of 5 × 10^3^ PFU mL^−1^ for 2 h before moving to experimental aquaria and performing the bacterial infection (MOI 1). In phage-coated sheet treatments, the plastic sheets (approx. 21 cm^2^, 3.3 × 10^5^—5.0 × 10^7^ PFU cm^−2^, manufactured and supplied by Fixed Phage Ltd, Glasgow, Scotland, UK) were placed into aquaria just before the addition of bacteria. In control treatments, Shieh medium replaced phage and/or bacteria and plastic sheets without phage-coating were applied. Survival of fish was monitored hourly for 24 h after addition of bacteria. Morbid fish that did not respond to stimuli were considered dead, removed from the experiment, and euthanised by decapitation. Bacterial cultivations from gills of all the dead fish were made on Shieh agar supplemented with tobramycin [54] to confirm the presence/absence of the pathogen. In experiment I, water samples (500 μL) for phage titer determination were taken from three replicate tanks per treatment at 0 h (right after the phage addition), 12 h, and 22 h (at the end of the experiment = 24 h after the bacterial infection) after the phage addition. At the end of the experiment, surviving fish were euthanised using 0.008% benzocaine.

### 4.5. Phage Therapy Experiment III: Phage Delivery in Flow-Through System

Water temperature in holding aquaria of fish was increased 1 °C day^−1^ from 15 to 20 °C, after which the fish were transferred to experimental aquaria. The experiment was started one day after transferring the fish, and the temperature increase continued one day after that by 1 °C day^−1^, from 20 to 25 °C.

Rainbow trout fry, averaging 3.03 g, were divided into 20 11-L flow-through aquaria supplied with aerated bore hole water. The 20 tanks were divided into five treatment groups, four aquaria with 48 fish in each (Table 4). The first group received phage lysate mix-coated (FCOV-F27 and FCL-2) feed (0.8 mm pellet size, 6 × 10^6^ PFU g^−1^), manufactured and supplied by Fixed Phage Ltd, Glasgow, Scotland, UK, daily for 7 days at 2% of body weight. Into three aquaria of the second fish group, phage mix-coated plastic sheets (A4 = 29.7 × 21.0 cm, 5 × 10^5^ PFU cm^−2^) were placed 7 days before bacterial infection (see below) for 2 weeks. One aquarium of the second group received a control sheet with no phage coating. One day before bacterial infection, a third group of fish (see below) was treated with a 2-h bath treatment in 9-L aquaria in aerated 25 °C bore hole water containing 1.5 × 10^6^ PFU mL^−1^ of phage mix, giving an MOI of 1. A fourth group received 1.5 × 10^6^ PFU mL^−1^ phage mix treatment right after the first symptoms of columnaris disease after bacterial infection was observed; the water flow from experimental aquaria was closed, phage mix solution added, and water flow opened again after 2 h. The fifth group of fish did not receive any phage treatment.

On day 8, three fish from three tanks of each treatment group were anesthetised with 0.008% benzocaine, and kidney, spleen, intestine, and skin mucus (carefully scraped with a scalpel from both sides of the fish) were collected for phage detection. At the same time, and also at the end of the experiment, three replicate water samples of 400 μL were taken from the same tanks. Water samples from one tank with only phage bath treatment were also taken after the experiment (Table 4). After the sampling on day 8, the fish from all the treatment groups were transferred to receive a 2-h bacterial infection in 9-L aquaria of aerated 25 °C bore hole water containing 1.5 × 10^6^ CFU mL^−1^ of a bacterium mix (FCO-F2 and B185). One tank per treatment received Shieh medium instead of bacteria. The exception was the treatment with the control plastic sheet that was also given a bacterial infection. The survival of fish was monitored for 6 days in 12 h intervals. Morbid fish that did not respond to stimuli were considered dead, removed from the experiment and euthanised by decapitation. Bacterial cultivations from gills of all the dead fish were made and live fish euthanised at the end of the experiment, as described above.

### 4.6. Phage Presence in Tissues Samples

Organs were aseptically removed, placed into 1.5 mL Eppendorf tubes, and smashed with plastic rods, after which 400 μL of Shieh medium was added. Skin mucus was transferred into Eppendorf tubes containing 200 μL of Shieh medium. Chloroform was added to organ, mucus, and water sample tubes at end concentration of 10%, tubes were vortexed and preserved at 7 °C for six days. Afterward, the samples were shortly centrifuged to separate chloroform and debris from the phage-containing Shieh phase that was collected and diluted tenfold in Shieh medium. Drop titrations (2 μL drops) were made on double-layer Shieh agar and plaques counted after two days to determine the presence of phages in the samples.

### 4.7. Statistical Analyses

Kaplan–Meier survival analysis (IBM SPSS Statistics version 24), log rank pairwise comparisons (Mantel–Cox), was used for analysis of cumulative mortality data.

### 4.8. Shelf-Life of Phages in Different Storage Conditions

Forty-six previously isolated phages [31] were used in the first shelf-life experiment (Table S1). High titer lysates (10^10^–10^11^ PFU mL^−1^) of phages were diluted into tenfold series with different buffers (2 mM Tris-HCl, MgCl_2_, and CaCl_2_) of three different pH (6.5, 7.5, and 8.0). Shieh medium (pH 7.5) was used as a control. Dilutions were applied on 96-well (1000 µL) storing plates, sealed with a parafilm, protected from light, and stored in a cold room (7 °C). Phage infectivity was tested after 1, 3, 5, 7, 14, 21, and 89 days: drop titrations (2 μL) were made on double-layer Shieh agar using the original host bacteria. The highest dilutions with clear plaques were recorded, and results expressed as an average of all the phages with the same original host.

Afterward, it was tested how phages used in therapy experiments (FCL-2, FCOV-S1, and FCOV-F27) tolerate different temperatures in different storage media. Phage titers were adjusted to 1 × 10^9^ PFU mL ^−1^ by diluting high titer lysates in 10 mL of Shieh medium (duplicate samples), autoclaved lake water (triplicate samples), or 0.9% NaCl (duplicate samples). Phage suspensions were stored at 21 °C, 7 °C, and −20 °C. Sampling for phage infectivity was conducted after 1, 4, 32, and 125 days by making tenfold dilutions from phage suspensions in original media. Samples in 0.9% NaCl, however, were diluted in autoclaved Milli-Q^®^ water. Suspensions preserved in −20 °C were thawed in a water bath (20 °C) before sampling. Drop titrations (2 μL) were made on double-layer Shieh agar and titers read after 2 days incubation in RT. We also tested if phages FCL-2, FCOV-S1, and FCOV-F27 could tolerate dehydration. Aliquots (500 µL) of phage lysates (in Shieh medium) were air-dried in a cold room (7 °C) in open Eppendorf tubes for two months, after which the tubes were closed. Phage infectivity was tested after 149 and 468 days by detaching a small amount of dry material from the bottom of the Eppendorf tube with 1 µL inoculation loop and suspending it to 200 µL of Shieh medium for PFU analysis.

## Figures and Tables

**Table 1:** *Flavobacterium columnare* and their phages used in this study. Bacteria and phages were isolated from Finnish and Swedish fish farms. *F. columnare* isolates have been previously categorised into genetic groups by restriction fragment length polymorphism analysis of the internal transcribed spacer region between 16S and 23S rRNA genes.

Bacterial Strain and Genetic Group	Phage Isolate and Genetic Group of the Host	Isolation Farm	Isolation Year	Experiment
FCO-S1 (A)		1	2017	I, II
FCO-F2 (C)		2	2017	I–III
B185 (G)		3	2008	I–III
	FCOV-S1 (A)	1	2017	I, II
	FCOV-F27 (C)	2	2017	I–III
	FCL-2 (G)	3	2008	I–III

**Table 2:** Setup of experiment I. Rainbow trout fry were infected individually with three *Flavobacterium columnare* isolates (FCO-S1, FCO-F2, and B185) (5 × 10^3^ CFU mL^−1^) and their mix. For phage treatment, purified phages (FCOV-S1, FCOV-F27, and FCL-2) (MOI 1) or their mix were added 2 h after and phage-coated plastic sheets (FCOV-F27, FCL-2, and control without phage coating) just before adding the bacteria. − = Shieh medium added instead of bacterium and/or phage.

Bacterial Isolate/mix	Phage Isolate/Mix	Fish n:o.	
FCO-S1	FCOV-S1	15	
FCO-S1	FCOV-F27	15	
FCO-S1	FCL-2	15	
FCO-F2	FCOV-S1	15	
FCO-F2	FCOV-F27	15	
FCO-F2	FCL-2	15	
B185	FCOV-S1	15	
B185	FCOV-F27	15	
B185	FCL-2	15	
Mix	FCOV-S1	15	
Mix	FCOV-F27	15	
Mix	FCL-2	15	
FCO-S1	Mix	15	
FCO-F2	Mix	15	
B185	Mix	15	
Mix	Mix	15	
FCO-F2	FCOV-F27-coated sheet	10	
B185	B185-coated sheet	10	
FCO-S1	−	10	No phage control
FCO-F2	−	10	No phage control
B185	−	10	No phage control
Mix	−	10	No phage control
−	FCOV-S1	10	No bacteria control
−	FCOV-F27	10	No bacteria control
−	FCL-2	10	No bacteria control
−	Control sheet	10	No bacteria control
−	Mix	10	No bacteria control
−	−	10	No treatment control
Total number of fish		360	

**Table 3:** Setup of experiment II. Rainbow trout fry were infected individually with three *Flavobacterium columnare* isolates (FCO-S1, FCO-F2, and B185) (5 × 10^3^ CFU mL^−1^) and their mix. For phage-treatment, a phage mix of three phages (FCOV-S1, FCOV-F27, and FCL-2) was added at MOI 1, 0.1, or 10 at the same time (0 h) or at MOI 1, 2 h after adding the bacteria. One group of fish was bath-exposed for 2 h to the phage mix (MOI 1) before transferring to experimental aquaria and adding the bacteria. − = Shieh medium added instead of bacterium and/or phage.

Bacterial Infection	Phage Treatment	Fish n:o
FCO-S1	MOI 1 0 h	15
FCO-S1	MOI 0.1 0 h	15
FCO-S1	MOI 10 0 h	15
FCO-S1	MOI 1 bath	15
FCO-S1	MOI 1 2 h	15
FCO-F2	MOI 1 0 h	15
FCO-F2	MOI 0.1 0 h	15
FCO-F2	MOI 10 0 h	15
FCO-F2	MOI 1 bath	15
FCO-F2	MOI 1 2 h	15
B185	MOI 1 0 h	15
B185	MOI 0.1 0 h	15
B185	MOI 10 0 h	15
B185	MOI 1 bath	15
B185	MOI 1 2 h	15
Mix	MOI 1 0 h	15
Mix	MOI 0.1 0 h	15
Mix	MOI 10 0 h	15
Mix	MOI 1 bath	15
Mix	MOI 1 2 h	15
FCO-S1	−	10
FCO-F2	−	10
B185	−	10
Mix	−	10
−	MOI 1 0 h	10
−	MOI 0.1 0 h	10
−	MOI 10 0 h	10
−	MOI 1-bath	10
−	MOI 1 2 h	10
−	−	10
Total number of fish		400

**Table 4:** Setup of experiment III. First group (feed) received feed coated with a phage mix (FCOV-F27 and FCL-2), daily for 7 days at 2% of body weight. Phage mix-coated plastic sheets were placed in tanks 7 days before bacterial infection for two weeks. One aquarium of the second group received a control sheet with no phage coating (control sheet). A third group of fish was placed one day before bacterial infection in a 2-h phage mix bath (pre-infection bath). The fish were infected with a bacterium mixture (FCO-F2 and B185) on day 8 in a bath for 2 h. A fourth group received a 2-h phage mix bath right after the first symptoms of columnaris disease after the bacterial infection were observed (bath post-infection). The fifth group of fish did not receive any phage mix treatment (control). Just before the bacterial infection, three fish from three tanks of each treatment group were anesthetised, and kidney, spleen, intestine, and skin mucus were collected for phage enumeration. At the same time and after the experiment, water samples were taken from the same tanks. + = bacterial infection/sampling for phage detection, − = no bacterial infection/no sampling for phage detection, ^a^ = only water sample after the experiment.

Delivery of Phage Mix	Bacterial Infection	Replicate	Fish n:o	Phage Sample
Feed	+	1	48	+
Feed	+	2	48	+
Feed	+	3	48	+
Feed	−	4	48	−
Sheet	+	1	48	+
Sheet	+	2	48	+
Sheet	+	3	48	+
Control sheet	+	4	48	−
Pre-infection bath	+	1	48	+
Pre-infection bath	+	2	48	+
Pre-infection bath	+	3	48	+
Pre-infection bath	−	4	48	−
Post-infection bath	+	1	48	+
Post-infection bath	+	2	48	+
Post-infection bath	+	3	48	+
Post-infection bath	−	4	48	+ ^a^
Control	+	1	48	+
Control	+	2	48	+
Control	+	3	48	+
Control	−	4	48	−

## Data Availability

The data of this manuscript is publicly available in JYX Digital Repository, https://doi.org/10.17011/jyx/dataset/77208 (accessed on 27 July 2021).

## Supplementary Materials

The following are available online at https://www.mdpi.com/article/10.3390/antibiotics10080914/s1, Figure S1: Shelf-life of *Flavobacterium columnare* phages in different buffers, Table S1: *Flavobacterium columnare* phages detected from fish organ and water samples in experiment III, Table S2: *Flavobacterium columnare* strains and phages used in phage shelf-life experiment with buffers.
