# Supplementary material for: Comparison of Delivery Methods in Phage Therapy against Flavobacterium columnare Infections in Rainbow Trout
Source: Antibiotics (Basel). 2021 Jul 27;10(8):914. doi: 10.3390/antibiotics10080914 (PMC8388691; doi:10.3390/antibiotics10080914)
Supplement: Supplementary file 1 [file antibiotics-10-00914-s001.zip › antibiotics-1291745-supplementary.pdf]

Supplementary material

Table S1. *Flavobacterium columnare* phages detected from fish organ and water samples in Experiment III. Samples from the organs were taken before the bacterial infection, water samples after the experiment. + phage detected, – = no bacterial infection, () = sample taken after the experiment, and i = sample inhibits bacterial growth. The number of + and i in phage columns indicate the number of replicate samples giving the particular result. Three fish were sampled from each replicate aquarium, + indicates positive results from an individual fish.

			**FCOV-F27**				**FCL-2**			
Delivery of phage mix	Bacterial infection	Replicate aquarium number	Water	Skin mucus	Intestine	Kidney	Water	Skin mucus	Intestine	Kidney
Feed	yes	1	i				i		+	ii
Feed	yes	2		+	+			+	+	ii
Feed	yes	3		+	+	+		+	+	
Sheet	yes	1								
Sheet	yes	2								
Sheet	yes	3								
Bath pre-infection	yes	1				i				
Bath pre-infection	yes	2	(+++)							
Bath pre-infection	yes	3	(+)			i				
Bath post-infection	yes	1	(+++)							
Bath post-infection	yes	2								
Bath post-infection	yes	3								
Bath post-infection	no	4				i	(+)			i
Control	yes	1								
Control	yes	2								
Control	yes	3				ii				i

Table S2. *Flavobacterium columnare* strains and phages used in phage shelf life experiment with buffers. Phages are grouped according to their host bacterium (genetic group of the bacterium in parentheses).

Host bacterium (genetic group)	B534 (A)	B537 (C)	FCO-F2 (C)	B185 (G)
Phage	FCOV-S1	FCOV-F1	FCOV-F10	FCOV-F13
		FCOV-F2	FCOV-F11	FCOV-F14
		FCOV-F3	FCOV-F18	FCOV-F15
		FCOV-F4	FCOV-F22	FCOV-F16
		FCOV-F5	FCOV-F24	FCOV-F45
		FCOV-F6	FCOV-F26	
		FCOV-F7	FCOV-F27	
		FCOV-F8	FCOV-F31	
		FCOV-F9	FCOV-F32	

FCOV-F12	FCOV-F39
FCOV-F17	FCOV-F40
FCOV-F19	FCOV-F41
FCOV-F20	FCOV-F42
FCOV-F21	FCOV-F43
FCOV-F23	FCOV-F44
FCOV-F25	
FCOV-F28	
FCOV-F29	
FCOV-F30	
FCOV-F33	
FCOV-F34	
FCOV-F35	
FCOV-F36	
FCOV-F37	
FCOV-F38	

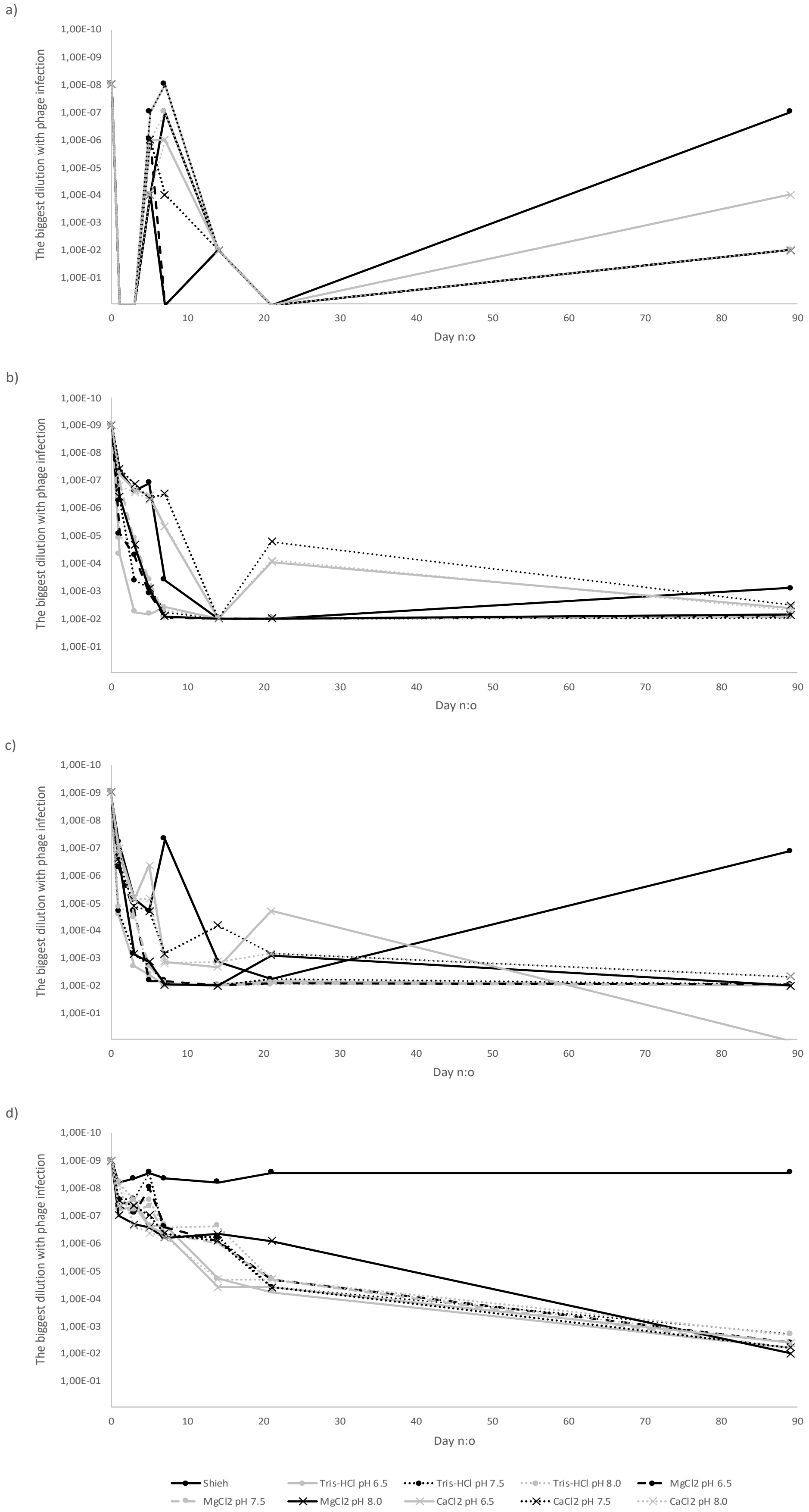

Figure S1. Shelf life (represented as the biggest dilution of phage sample able to infect host bacterium) of *Flavobacterium columnare* phages preserved in different buffers and infecting different host bacteria belonging to genetic groups a) A (host bacterium B534), b) C (host bacterium B537), c) C (host bacterium FCO-F2) and d) G (host bacterium B185).
